# Diagnostic value of serum HER2 levels in breast cancer: a systematic review and meta-analysis

**DOI:** 10.1186/s12885-020-07545-2

**Published:** 2020-10-31

**Authors:** Amir Shamshirian, Amir Reza Aref, George W. Yip, Majid Ebrahimi Warkiani, Keyvan Heydari, Sajad Razavi Bazaz, Zeinab Hamzehgardeshi, Danial Shamshirian, Mahmood Moosazadeh, Reza Alizadeh-Navaei

**Affiliations:** 1 Department of Medical Laboratory Sciences, Student Research Committee, School of Allied Medical Science, Mazandaran University of Medical Sciences, Sari, Iran; 2 Gastrointestinal Cancer Research Center, Non-Communicable Diseases Institute, Mazandaran University of Medical Sciences, Sari, Iran; 3 Belfer Center for Applied Cancer Science, Department of Medical Oncology, Dana-Farber Cancer Institute, Harvard Medical School, Boston, MA 02215 USA; 4 Department of Anatomy, Yong Loo Lin School of Medicine, National University of Singapore, Singapore, 117594 Singapore; 5 School of Biomedical Engineering, University of Technology Sydney, Sydney, Ultimo, NSW 2007 Australia; 6 Institute of Molecular Medicine, Sechenov First Moscow State University, Moscow, 119991 Russia; 7 Student Research Committee, School of Medicine, Mazandaran University of Medical Sciences, Sari, Iran; 8 Sexual and Reproductive Health Research Center, Mazandaran University of Medical Sciences, Sari, Iran; 9 Chronic Respiratory Diseases Research Center, National Research Institute of Tuberculosis and Lung Diseases (NRITLD), Shahid Beheshti University of Medical Sciences, Tehran, Iran; 10 Health Sciences Research Center, Addiction Institute, Mazandaran University of Medical Sciences, Sari, Iran

**Keywords:** Serum HER-2, Breast neoplasm, Immunoassay, *erb*B2

## Abstract

**Background:**

Measurement of serum human epidermal growth factor receptor-2 (HER-2/*neu*) levels might play an essential role as a diagnostic/screening marker for the early selection of therapeutic approaches and predict prognosis in breast cancer patients. We aimed to undertake a systematic review and meta-analysis focusing on the diagnostic/screening value of serum HER-2 levels in comparison to routine methods.

**Methods:**

We performed a systematic search via PubMed, Scopus, Cochrane-Library, and Web of Science databases for human diagnostic studies reporting the levels of serum HER-2 in breast cancer patients, which was confirmed using the histopathological examination. Meta-analyses were carried out for sensitivity, specificity, accuracy, area under the ROC curve (AUC), positive predictive value (PPV), negative predictive value (NPV), positive likelihood ratio (PLR), and negative likelihood ratio (NLR).

**Results:**

Fourteen studies entered into this investigation. The meta-analysis indicated the low sensitivity for serum HER2 levels (Sensitivity: 53.05, 95%CI 40.82–65.28), but reasonable specificity of 79.27 (95%CI 73.02–85.51), accuracy of 72.06 (95%CI 67.04–77.08) and AUC of 0.79 (95%CI 0.66–0.92). We also found a significant differences for PPV (PPV: 56.18, 95%CI 44.16–68.20), NPV (NPV: 76.93, 95%CI 69.56–84.31), PLR (PLR: 2.10, 95%CI 1.69–2.50) and NLR (NLR: 0.58, 95%CI 0.44–0.71).

**Conclusion:**

Our findings revealed that although serum HER-2 levels showed low se nsitivity for breast cancer diagnosis, its specificity, accuracy and AUC were reasonable. Hence, it seems that the measurement of serum HER-2 levels can play a significant role as a verification test for initial negative screening test results, especially in low-income regions due to its cost-effectiveness and ease of implementation.

**Supplementary Information:**

The online version contains supplementary material available at 10.1186/s12885-020-07545-2.

## Background

The most common deadly cancer among women is breast cancer throughout the world; this condition is more severe in developing countries [1, 2]. As a heterogeneous complex disease, this malignancy includes different subtypes with different clinical outcomes and treatment responses [3]. Previous investigations showed that the early detection and diagnosis of this malignancy could lead to promising treatment and improve the chance of successful therapy [4]. In this regard, there are different types of diagnostic approaches for the detection of breast cancer, such as magnetic resonance imaging (MRI) of the breast, mammography, molecular imaging, biopsy, and ultrasound tomography [5].

The human epidermal growth factor receptor-2 (HER-2/*neu*) or *c-erbB-2/neu* is one of the epidermal growth factor receptor (EGFR) family members. As an oncogene, the amplification of HER-2 or its protein overexpression has a significant role in the development of malignant types of breast cancer, which observes in 20–30% of breast cancer patients [6, 7]. Remarkably, Slamon et al. [8] have understood the importance of HER-2 as a diagnostic factor for breast cancer in 1987. Recently, this protein has gained significant attention as a biomarker as well as a target of diagnosis, prognosis, and therapy in breast cancer patients [9].

There are several methods to determine the presence of HER-2, which are 1. gene copy measuring by polymerase chain reaction (PCR), Southern blot analysis, Chromogenic In Situ Hybridization (CISH) and Fluorescence In Situ hybridization (FISH); 2. messenger RNA measuring by PCR or Southern blot analysis; 3. protein expression measuring by Western blot and Immunohistochemical (IHC) analysis; and 4. serum antigen measuring by Enzyme-Linked Immunosorbent Assay (ELISA) or chemiluminescence immunoassays (CLIA). It is highly recommended to use IHC for the evaluation of HER2 status. In this regard, IHC scored 3+ would be considered as positive status and 0/1+ as a negative status. In this way, score 2+ is uncertain and should be confirmed through FISH as a gold standard [10, 11].

The HER-2 protein contains three different domains, including transmembrane, extracellular, and intracellular tyrosine kinase domain. The extracellular domain (ECD) can be released into the blood after cleavage and shedding from the tumor cell surface by metalloproteases [12, 13]. Therefore, it can be detected in the serum. Serum HER-2 levels increase in 18% of primary breast cancers and 46% of metastatic breast cancers [14, 15]. There are several shreds of evidence regarding the correlation of serum HER-2 levels and tissue HER-2 protein overexpression as well as poor prognosis in a metastatic type of breast cancer [16–19].

Nevertheless, the most effective method for HER-2 measurement is controversial in the case of efficacy and ease of implementation [7]. In fact, several discrepancies between utilization of serum or tissue HER2 have raised to the researchers in the field such as a shortage in defining tissue positivity for HER2 due to classification system, heterogeneity of breast cancers, conversion of HER2 status and the kinetic nature of serum HER2 ECD concentrations, cut-off levels, serum interference, etc. [20]. Following such disagreements, measurement of serum HER-2 level is not recommended in any clinical procedure according to the “*American Society of Clinical Oncology (ASCO) 2007 update of recommendations for the use of tumor markers in breast cancer*” [21].

However, over time, it seems that HER-2 measuring can play an important role as a diagnostic marker or at least screening marker for the early selection of therapeutic approaches as well as predict prognosis in breast cancer patients. In fact, any carelessness regarding HER2 status can change the treatment approach. Regardless of therapies’ side effects, it will have a high-cost burden on patients and endangering their lives. Hence, we aimed to undertake a systematic review and meta-analysis focusing on the diagnostic/screening values of serum HER-2 levels compared to reference methods of FISH/IHC due to its ease of application and cost-effectiveness. We hope our findings could help the controversies on the subject and be useful for the new update of recommendations for the use of tumor markers in breast cancer.

## Method

### Search strategy

In order to study design, search strategy, screening, and reporting, we followed the Preferred Reporting Items for Systematic Reviews and Meta-Analyses (PRISMA) guidelines. We performed a systematic search via PubMed, Scopus, Cochrane Library, and Web of Science databases up to 15 February 2019. The search strategy included MeSH terms and free keywords as follows: ((Breast OR Mammary) AND (Cancer* OR Neoplasm* OR Tumor* OR Malignancy* OR Carcinoma*) AND (HER2* OR erbB2*) AND (Sensitivity* OR Specificity*) AND Serum). Our search was restricted to English papers, but there was no limitation regarding the date of publications. Only human diagnostic studies on breast cancer without criteria for the types were included.

### Criteria study selection

Two members of our group (A.SH and R.AN) selected the studies independently and discussed to solve the disagreements. Inclusion and exclusion criteria are presented in Table 1.

**Table 1:** Eligibility criteria

Inclusion criteria	Exclusion criteria
• human diagnostic studies reported the level of serum HER2 in breast cancer patients	• Non-human subjects
• Studies confirmed the breast cancer using immunohistopathological examination	• Conference abstracts
• Studies reported the sensitivity and specificity of serum HER2 level or comprised with data could to calculate the desiered parameters mentioned in data extraction section	• Grey literature
	• Comments
• Full text published papers	• Letters
• English language	• Reviews
	• Case reports
	• In vitro studies
	• Ecological studies
	• Duplicate publications
	• Studies with insufficient data

### Data extraction & quality assessment

Two investigators (A.SH and K.HD) have independently assessed the quality of studies and extracted data from included papers. The supervisor (R.AN) resolved any disagreements in this part. Data extraction checklist included the name of first author, publication year, number of patients, mean age, histopathological results, serum HER2 level, area under the ROC Curve (AUC), true positives (TP), true negatives (TN), false positives (FP) and false negatives (FN) of serum HER2 level, clinicopathological features, and available correlations. To assess the quality of included studies, Quality Assessment of Diagnostic Accuracy Studies (QUADAS-2) tool was used.

### Data analysis

Sensitivity, specificity, positive predictive values (PPV), negative predictive values (NPV), positive likelihood ratio (PLR), negative likelihood ratio (NLR), and accuracy and 95% confidence interval were calculated with Medcalc. Statistical analysis was performed using STATA v.11 software. To assess the heterogeneities, we used *I*-square (*I*^*2*^) test. According to extreme heterogeneity, the random-effects model was used for the calculation of pooled estimation. For the finding of suspected parameters for heterogeneity, we used sensitivity analysis as well for serum level cut-off. The possible publication bias was evaluated using Egger’s asymmetry test, which presented by the funnel plot. *P*-values less than 0.05 were considered statistically significant.

### Ethical approval

Study protocol has been registered in the International Prospective Register of Systematic Reviews (PROSPERO) due to code CRD42019126703.

## Results

### Study selection process

Our initial database search contained 1066 papers. After removing duplicated articles, we used the title and abstract for screening remaining studies. Finally, 77 papers considered for eligibility assessment, of which 14 studies entered into the meta-analysis. The PRISMA flow diagram for the study selection process is presented in Supplementary Fig. 1.

### Study characteristics

Out of selected studies, a total of 3528 breast cancer patients with age ranged between 25 to 93 were included in our study. The cut-off value was set at 15.0 ng/ml for serum-HER2 concentration in most of the studies, according to the Food and Drug Administration (FDA) and various manufacturer’s recommendations for breast cancer. Seven studies used CLIA, five studies used ELISA, and two studies used both methods for the determination of serum-HER2 levels. Characteristics of studies entered into meta-analysis are presented in Table 2.

**Table 2:** Characteristics of studies entered into the meta-analysis

Author	Year	Country	No. of cases	Reference method	Detection method	Cut-off value
Kong [17]	2006	Korea	195	IHC/FISH	CLIA	15 μg/L
Olsen [22]	2007	Germany	118	IHC/FISH	ELISA/ CLIA	15 ng/mL
Ludovini [23]	2008	Italy	256	IHC/FISH	ELISA/ CLIA	15 ng/mL
Papadopoulou [24]	2008	Greece	56	^a^	ELISA	1.98 ng/ml
Savino [25]	2009	Italy	85	IHC	ELISA	22 ng/mL
Finn [26]	2009	–	579	IHC/FISH	ELISA	16 ng/mL
Farzadnia [27]	2010	Iran	75	IHC	ELISA	18.4 ng/ml
Lauterlein [28]	2011	Denmark	311	IHC/FISH	CLIA	12.2 mg/L
Sørensen [29]	2013	Denmark	540	IHC/FISH	CLIA	15 μg/L
Pedersen [30]	2013	Denmark	107	IHC/FISH	CLIA	15 μg/L
Di Gioia [31]	2014	Germany	565	IHC/FISH	CLIA	15 ng/mL
Di Gioia [32]	2015	Germany	241	IHC/FISH	CLIA	15 ng/mL
Banys-Paluchowski [33]	2017	Germany	251	IHC/FISH	ELISA	15 ng/mL
Broughton [34]	2017	Norway	149	IHC/FISH	CLIA	15.2 μg/L

*IHC* Immunohistochemistry, *FISH* Fluorescence in situ hybridization, *CLIA* Chemiluminescence immunoassay, *ELISA* Enzyme-linked immunosorbent assay, ^a^ only mentioned histological examination

### Quality assessment

According to quality assessment using the QUADAS-2 tool, 14 papers earned the eligibility score and entered into the meta-analysis. The quality assessment graph and methodological quality summary are presented in Supplementary Fig. 2 and 3.

### Egger’s test

Egger’s test indicated a significant publication bias for serum sensitivity (*P* = 0.001), specificity (*P* = 0.009), PPV (*P* = 0.019), and NLR (*P* = 0.001). Publication bias for NPV (*P* = 0.073), PLR (*P* = 0.084), accuracy (*P* = 0.086), and AUC was not significant (*P* = 0.169).

### Main outcomes

#### Sensitivity (Fig. 1)

Carrying meta-analysis on 14 studies indicated a low sensitivity for serum-HER2 measurement in comparison to histopathological examinations (Sensitivity: 53.05, 95%CI 40.82–65.28). Significant heterogeneity was observed (*I*^*2*^ = 94.0%, *P* < 0.0001). Sensitivity analysis using the same cut-offs indicated no significant deferences (Sensitivity: 51.49, 95%CI 35.27–67.71) (Supplementary Fig. 4).

**Fig. 1:**
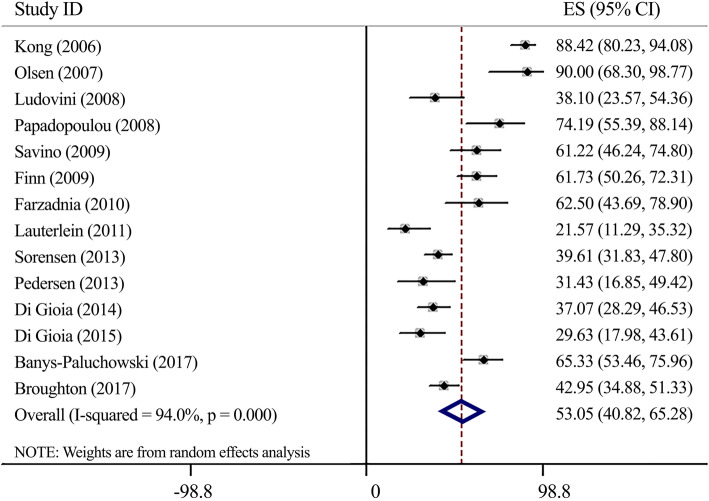
Forest plot for serum-HER2 sensitivity

#### Specificity (Fig. 2)

According to meta-analysis of 13 papers’ outcomes, findings revealed a substantial specificity for the measurement of serum-HER2 in breast cancer diagnosis (Specificity: 79.27, 95%CI 73.02–85.51). The heterogeneity was considerable (*I*^*2*^ = 93.8%, *P* < 0.0001). Sensitivity analysis using the same cut-offs indicated no significant deferences (Specificity: 81.52, 95%CI 73.48–89.56) (Supplementary Fig. 5).

**Fig. 2:**
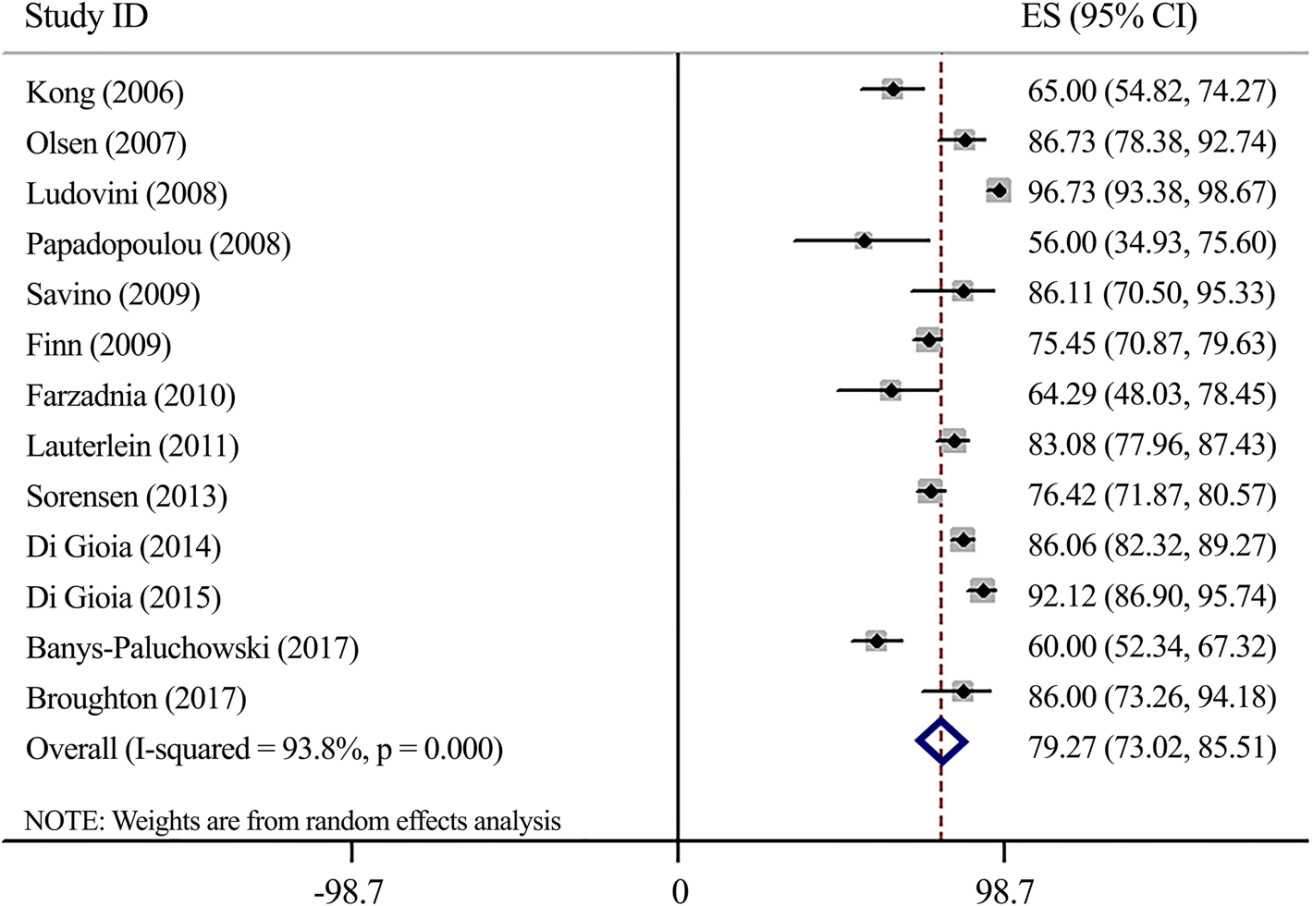
Forest plot for serum-HER2 specificity

#### PPV & NPV (Fig. 3)

Considering the performance of a diagnostic test, our meta-analysis demonstrated the PPV of 56.18 (95%CI 44.16–68.20) and the NPV of 76.93 (95%CI 69.56–84.31) for serum-HER2 examination. The heterogeneity was significant for both PPV and NPV (*I*^*2*^ = 94.4%, *P* < 0.0001, *I*^*2*^ = 98.5%, *P* < 0.0001, respectively). Sensitivity analysis using the same cut-offs indicated no significant deferences for both PPV (PPV: 58.37, 95%CI 43.74–73.00) (Supplementary Fig. 6) and NPV (NPV: 78.08, 95%CI 67.19–88.96) (Supplementary Fig. 7).

**Fig. 3:**
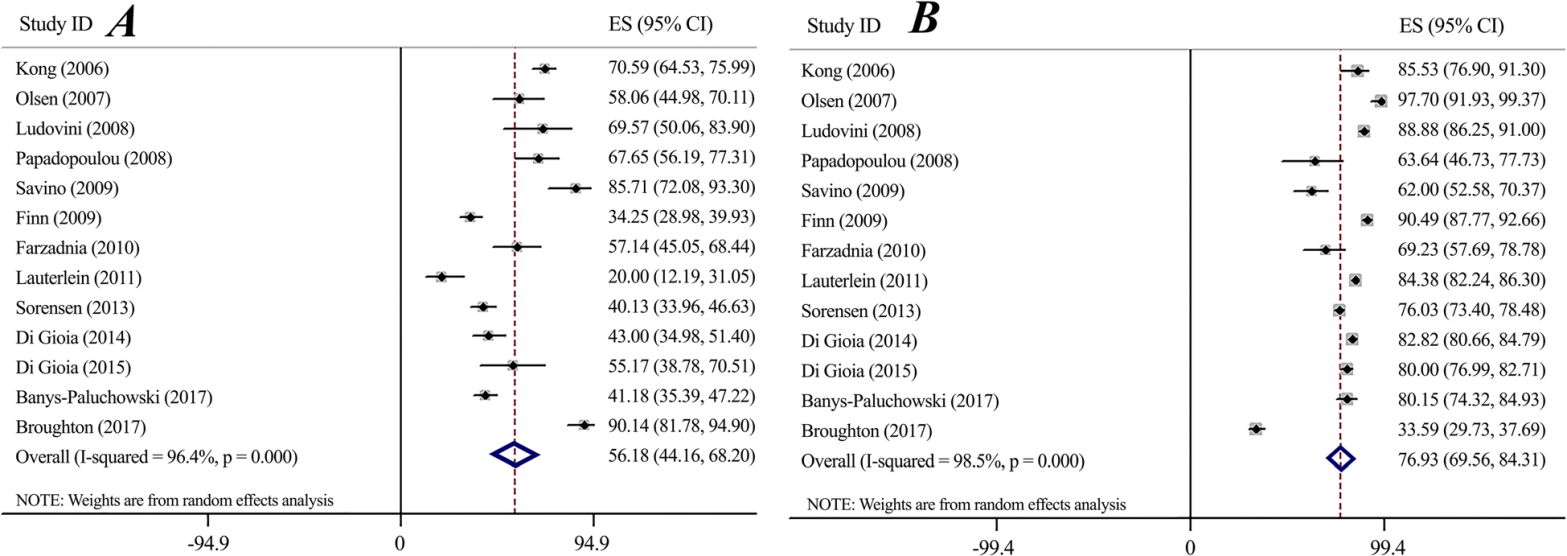
Forest plot for serum-HER2 Positive Predictive Value (A) and Negative Predictive Value (B)

#### PLR & NLR (Fig. 4)

Regarding value of performing a diagnostic test, meta-analysis found the significant PLR 2.10 (95%CI 1.69–2.50) and NLR 0.58 (95%CI 0.44–0.71) for the serum-HER2 test. The high heterogeneity was observed for both PLR and NLR (*I*^*2*^ = 57.7%, *P* = 005, *I*^*2*^ = 90.6%, *P* < 0.0001, respectively). Sensitivity analysis using the same cut-offs indicated no significant deferences for both PLR (PLR: 2.33, 95%CI 1.72–2.95) (Supplementary Fig. 8) and NLR (NLR: 0.56, 95%CI 0.38–0.74) (Supplementary Fig. 9).

**Fig. 4:**
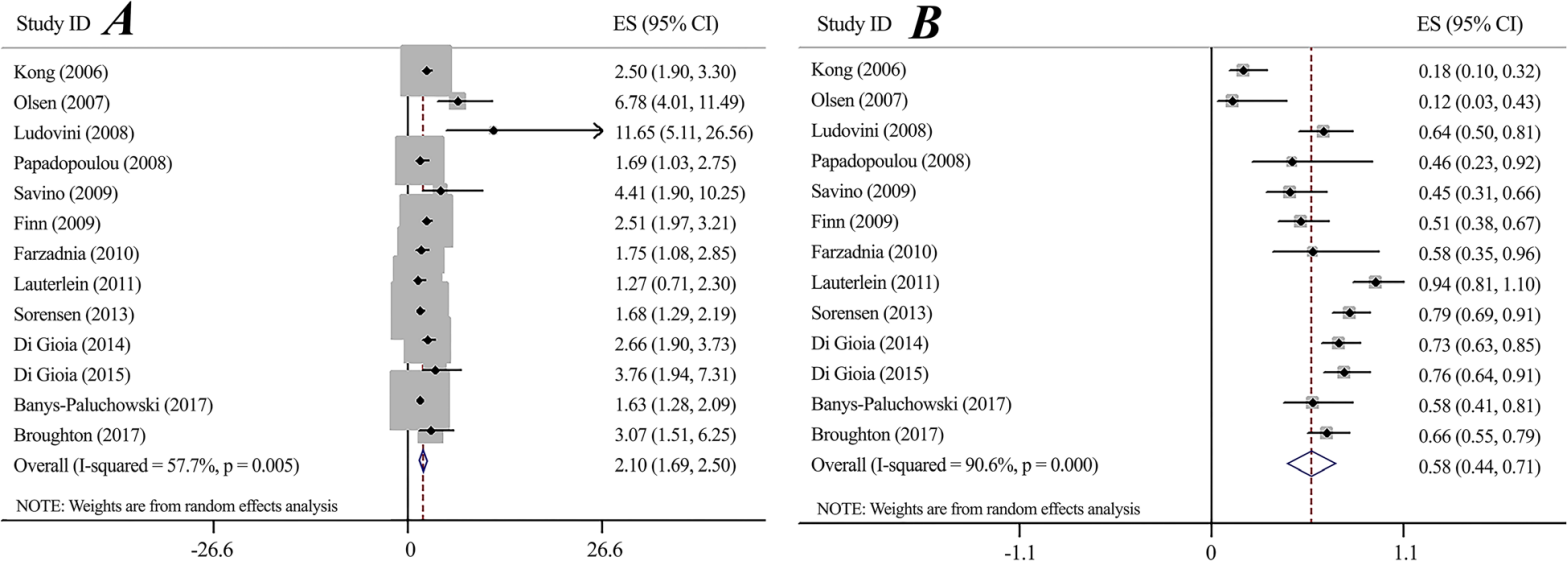
Forest plot for serum-HER2 Positive Likelihood Ratio (A) and Negative Likelihood Ratio (B)

#### Accuracy (Fig. 5)

By examining sensitivity and specificity, we found the accuracy of 72.06 (95%CI 67.04–77.08) for this biomarker. Substantial heterogeneity was observed (*I*^*2*^ = 90.0%, *P* < 0.0001). Sensitivity analysis using the same cut-offs indicated no significant deferences (Accuracy: 73.12, 95%CI 56.68–80.56) (Supplementary Fig. 10).

**Fig. 5:**
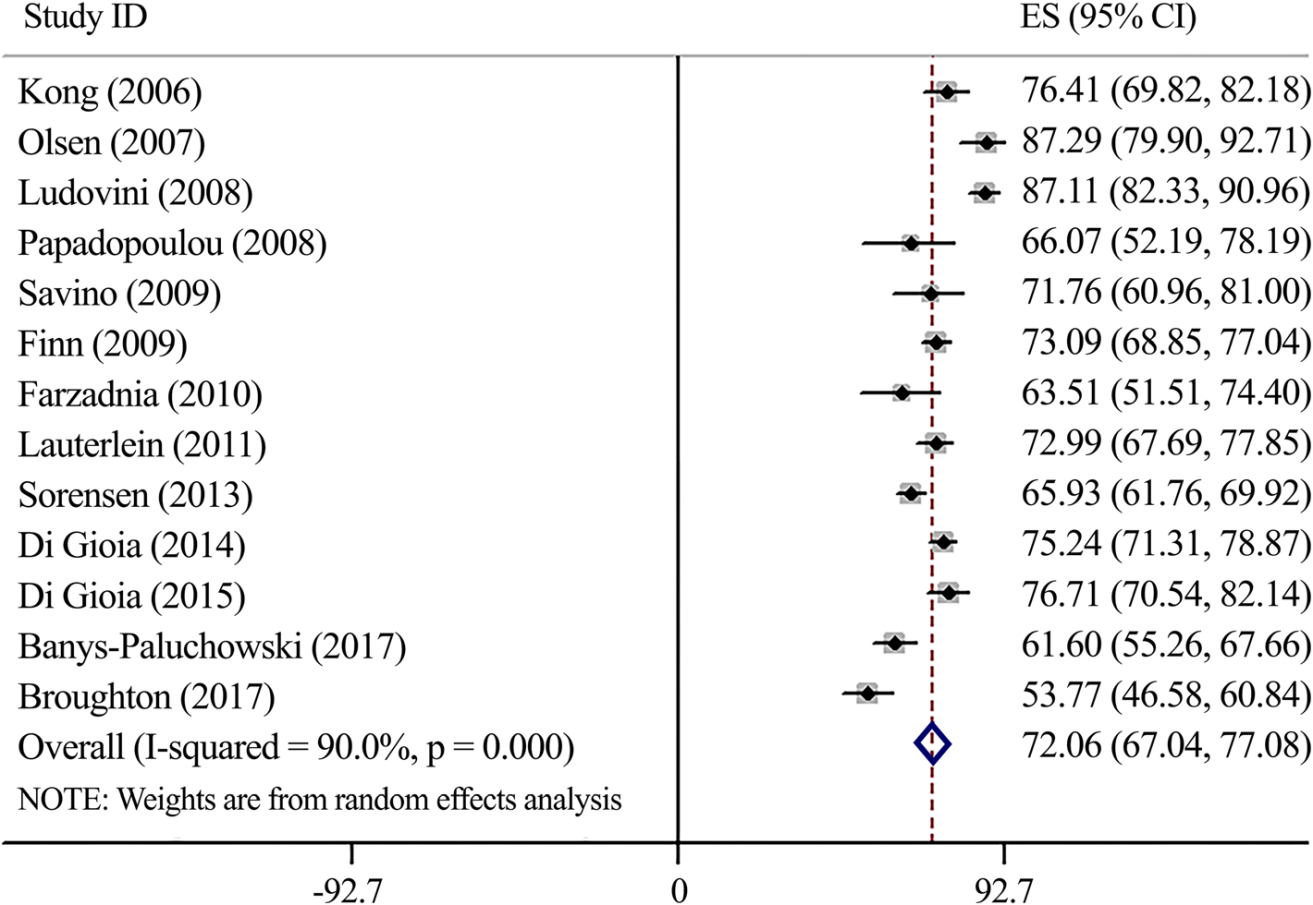
Forest plot for serum-HER2 accuracy

### Area under the ROC curve (Fig. 6)

Considering the AUCs that could be extracted only from three studies, meta-analyses of AUCs resulted in overall AUC of 0.79 (95%CI 0.66–0.92). The heterogeneity was significant (*I*^*2*^ = 87.6%, *P* < 0.0001).

**Fig. 6:**
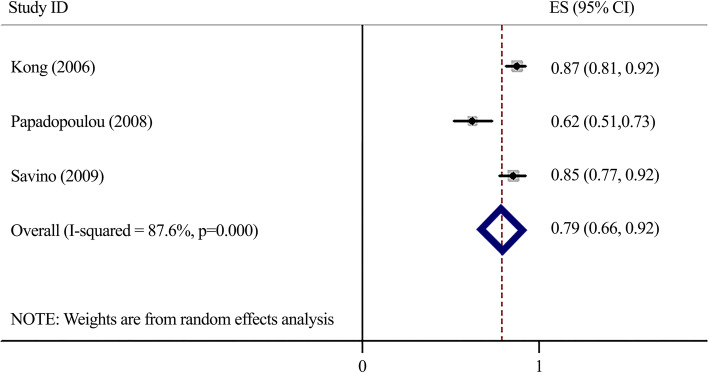
Forest plot for Area under the ROC Curve

### Meta-regression analysis

To find the impact of moderator variables on heterogeneities, meta-regression analysis considering publication year (*P* = 0.31), mean age (*P* = 0.72), and metastatic/non-metastatic condition (*P* = 0.79) resulted in no significant relationships.

## Discussion

According to all controversies regarding the diagnostic/screening value of serum-HER2 in breast cancer, to overcome this debate, we conducted a meta-analysis to integrate the results of studies included with adequate data for calculation of the test’s accuracy in this regard. Finally, we found that serum-HER2 indicated a high specificity for detecting tissue HER2 status in breast cancer. In detail, specificity for a screening test can be defined as the test’s ability to identify the true negative cases or, in other words, identifying all cases which do not have the target disorder based on the performance relative to a gold standard [35]. Accordingly, our meta-analysis indicated the specificity 79.27 (95%CI 73.02–85.51) for the serum-HER2 test, the test’s ability to detect individuals with the negative result based on the presence/absence of breast cancer is high and satisfactory.

In order to apply appropriate treatments for a disorder, we mainly need to more accurate diagnostic tests, especially with the development of modernity toward higher speed, cost-effectiveness, performance, and safety, which resulted in several available diagnostic tests for a particular condition [36]. Focusing on our study, in several cancers, HER2 status is a necessary item for HER2 targeted therapy, which mostly will be determined using biopsy specimens. The biopsy problem is that it is not always easily available, and the expression of HER2 is heterogeneous within tumor tissue, which might lead to a false negative outcome [37]. Also, given our experiences, other limitations might affect the results as follows: 1. The lab is not equipped for running this test; 2. The cancer is HER2 negative; and 3. The tumor is small, and therefore, the amount of HER2 that is shed into the bloodstream is limited.

Among all available HER-2 examination methods, IHC and FISH were the most preferred techniques among clinicians and researchers [38]. However, the desire for the measurement of serum-HER2 levels as a non-invasive technique for breast cancer diagnosis and prognosis has attracted the investigators’ interests in two recent decades [39, 40].

In more detail, sensitivity for a screening test can be defined as the test’s ability to identify the true positive cases or, in other words, identifying all cases with the considered disorder based on performance relative to a gold standard [35]. Accordingly, our findings showed a sensitivity of 53.05 (95%CI 40.82–65.28) for the serum-HER2 test; the ability of the test for detection of individuals with a positive result based on reference standard is low and unsatisfactory.

From another point of view, the indicator PPV demonstrates that how many people tested positive based on the screening test are actually have the considered condition [35]. In this regard, our meta-analysis showed the PPV 56.18 (95%CI 44.16–68.20), which is low as like as sensitivity for the serum-HER2 test and suggest unsatisfactory performance for this test in comparison to the gold standard.

Moreover, the indicator NPV indicates that how many people tested negative based on screening tests truly do not have the target condition. In this regard, our findings showed the NPV 76.93 (95%CI 69.56–84.31), which is high as like as specificity for the serum-HER2 test and suggest satisfactory performance for this test in comparison to the gold standard.

Altogether, owing mentioned metrics, another metric is proposed as a diagnostic test accuracy or effectiveness, which is defined as the test’s ability to differentiate between subjects with or without the target disorder. In other words, the test’s ability to classify true positive and negative subjects among all subjects [41, 42]. Our results revealed the accuracy of 72.06 (95%CI 67.04–77.08) for the serum-HER2 test, which is reasonably high and suggests satisfactory performance in this regard.

It is worth mentioning that the prevalence of the target condition affects the diagnostic accuracy of the test. If the sensitivity and specificity do not change, the accuracy of the test will increase by decreasing condition prevalence [43].

As an alternative statistic, positive and negative likelihood ratios are powerful metrics for diagnostic accuracy summarising [44]. The likelihood ratio defines as the probability of test results in subjects with the condition to the probability in the subjects without the condition [45]. In this regard, our findings demonstrated that PLR 2.10 (95%CI 1.69–2.50) and NLR 0.58 (95%CI 0.44–0.71) for the serum-HER2 test, which shows an association with the presence and absence of the condition, respectively. However, it is reported that PLR greater than 10 and NLR less than 0.1 provide strong evidence for diagnosis [46].

It is significant that if the entered studies into meta-analysis use different cut-off values for presenting positive results for a test, the threshold effect will arise toward bias [47]. We conducted the sensitivity analysis on studies with the same cut-off values to avoid the bias, which revealed no significant differences (Supplementary File).

As far as we know, this meta-analysis demonstrates the first report of evaluating the diagnostic/screening values of serum-HER2 in breast cancer patients. In this regard, Zhang et al. [48] study has indicated a high specificity and moderate diagnostic value for serum-HER2 in gastric cancer patients as a potential surrogate biomarker of HER2 status.

Considering all the facts mentioned above, although the serum-HER2 test has failed to be considered as a gold standard test, according to the shreds of evidence, this test can be beneficial for the detection of true negatives (HER2 negative status, or absence of breast cancer), especially in low-income regions due to its cost-effectiveness and ease of implementation. However, further extensive prospective studies are needed to robust the findings of this study. Also, monitoring serum-HER2 concentrations during treatment and tumor progression is recommended to find the prognosis values of serum-HER2 in such patients.

Given the fact that limitations are unavoidable and all studies face that, as a potential limitation in the present study, although most studies used the same cut-off values since we had no access to raw data, we could not define an ideal threshold for the serum-HER2 test in the diagnosis of breast cancer.

## Conclusion

As far as we know, this systematic review and meta-analysis is the first report regulated numerical data in order to find the diagnostic values of serum-HER2 test in breast cancer. Our findings indicated that, although serum HER2 levels showed low sensitivity for breast cancer diagnosis, its specificity is significantly high. Hence, it seems that the measurement of serum HER2 levels can play a significant role as a verification test for initial negative screening test results, especially in low-income regions due to its cost-effectiveness and ease of implementation.

## Supplementary Information

**Additional file 1 Supplementary Fig. 1**. PRISMA Flowchart of the study selection procedure. **Supplementary Fig. 2**. Quality assessment graph. **Supplementary Fig. 3**. methodological quality summary. **Supplementary Fig. 4**. Forest plot for sensitivity analysis of serum-HER2 sensitivity. **Supplementary Fig. 5**. Forest plot for sensitivity analysis of serum-HER2 specificity. **Supplementary Fig. 6**. Forest plot for sensitivity analysis of serum-HER2 Positive Predictive Value. **Supplementary Fig. 7**. Forest plot for sensitivity analysis of serum-HER2 Negative Predictive Value. **Supplementary Fig. 8**. Forest plot for sensitivity analysis of serum-HER2 Positive Likelihood Ratio. **Supplementary Fig. 9**. Forest plot for sensitivity analysis of serum-HER2 Negative Likelihood Ratio. **Supplementary Fig. 10**. Forest plot for sensitivity analysis of serum-HER2 accuracy.

## Data Availability

Not applicable.

## Abbreviations

HER-2/*neu*: Human epidermal growth factor receptor-2
AUC: Area under the ROC curve
PPV: Positive predictive value
NPV: Negative predictive value
PLR: Positive likelihood ratio
NLR: Negative likelihood ratio
MRI: Magnetic resonance imaging
EGFR: Epidermal growth factor receptor
PCR: Polymerase chain reaction
CISH: Chromogenic in situ hybridization
FISH: Fluorescence in situ hybridization
IHC: Immunohistochemical
ELISA: Enzyme-linked immunosorbent assay
CLIA: Chemiluminescence immunoassays
ECD: Extracellular domain
ASCO: American society of clinical oncology
PRISMA: Preferred reporting items for systematic reviews and meta-analyses
TP: True positives
TN: True negatives
FP: False positives
FN: False negatives
QUADAS-2: Quality assessment of diagnostic accuracy studies
PROSPERO: International prospective register of systematic reviews
FDA: Food and drug administration
