# Supplementary material for: Diagnostic value of serum HER2 levels in breast cancer: a systematic review and meta-analysis
Source: BMC Cancer. 2020 Oct 31;20:1049. doi: 10.1186/s12885-020-07545-2 (PMC7603697; doi:10.1186/s12885-020-07545-2)
Supplement: Supplementary file 1 — Additional file 1 Supplementary Fig. 1. PRISMA Flowchart of the study selection procedure. Supplementary Fig. 2. Quality assessment graph. Supplementary Fig. 3. methodological quality summary. Supplementary Fig. 4. Forest plot for sensitivity analysis of serum-HER2 sensitivity. Supplementary Fig. 5. Forest plot for sensitivity analysis of serum-HER2 specificity. Supplementary Fig. 6. Forest plot for sensitivity analysis of serum-HER2 Positive Predictive Value. Supplementary Fig. 7. Forest plot for sensitivity analysis of serum-HER2 Negative Predictive Value. Supplementary Fig. 8. Forest plot for sensitivity analysis of serum-HER2 Positive Likelihood Ratio. Supplementary Fig. 9. Forest plot for sensitivity analysis of serum-HER2 Negative Likelihood Ratio. Supplementary Fig. 10. Forest plot for sensitivity analysis of serum-HER2 accuracy. [file 12885_2020_7545_MOESM1_ESM.docx]

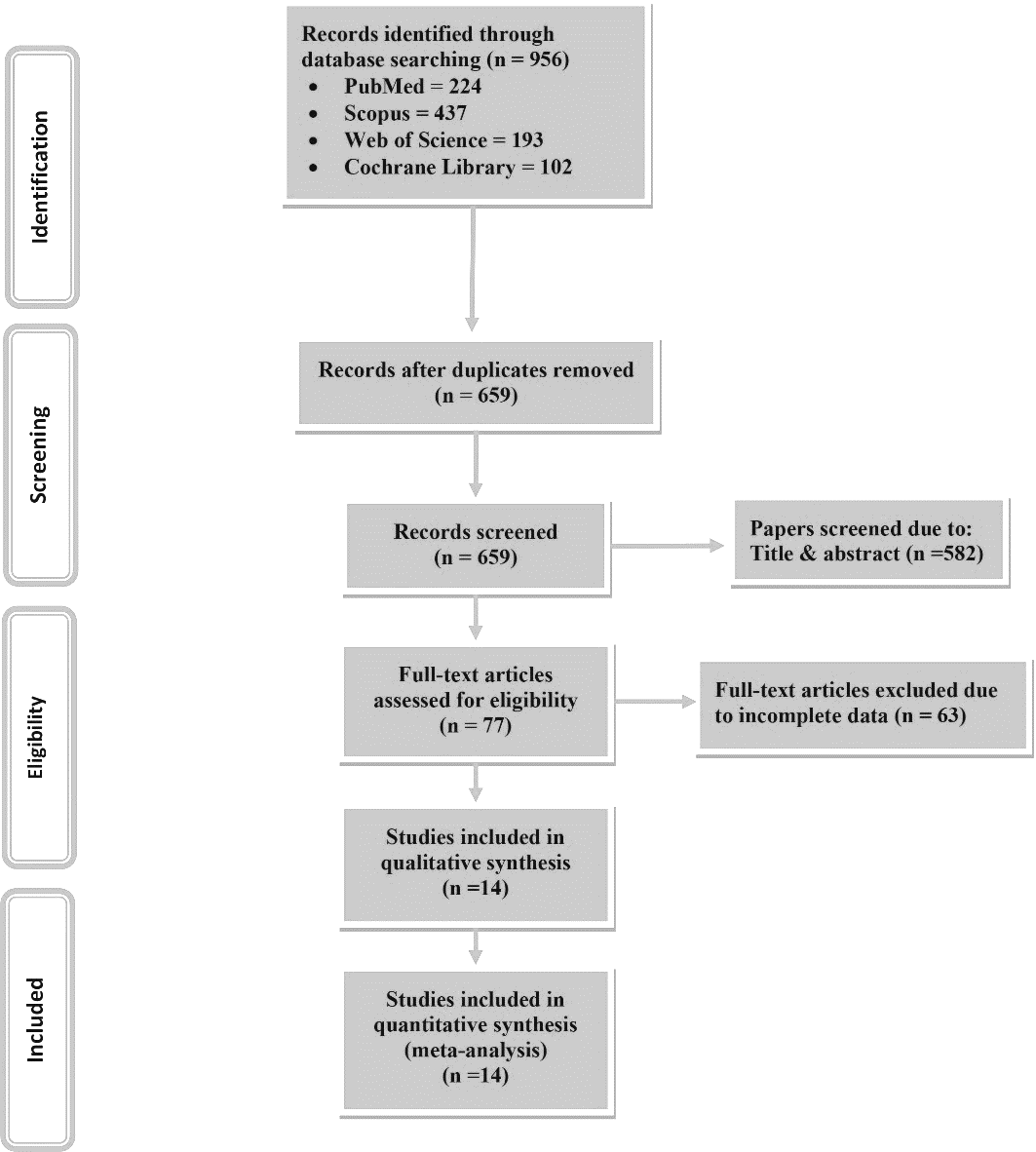


Supplementary Figure 1. PRISMA Flowchart of the study selection procedure


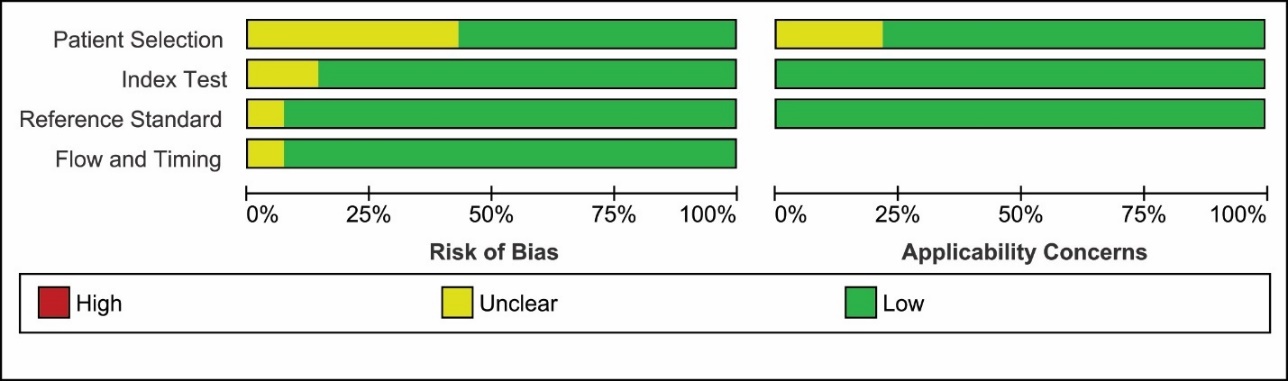


Supplementary Figure 2. Quality assessment graph

*
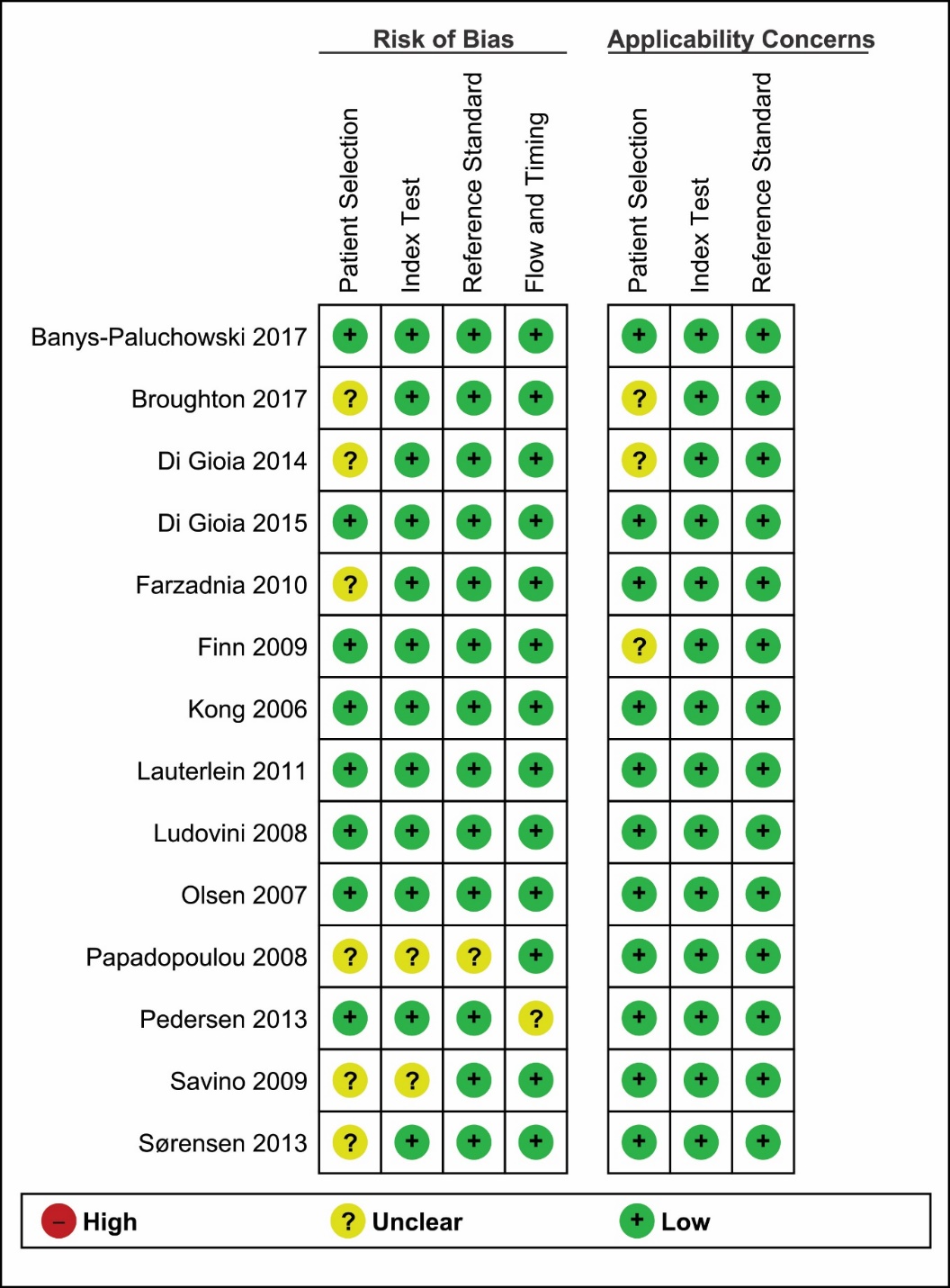
*

Supplementary Figure 3. methodological quality summary


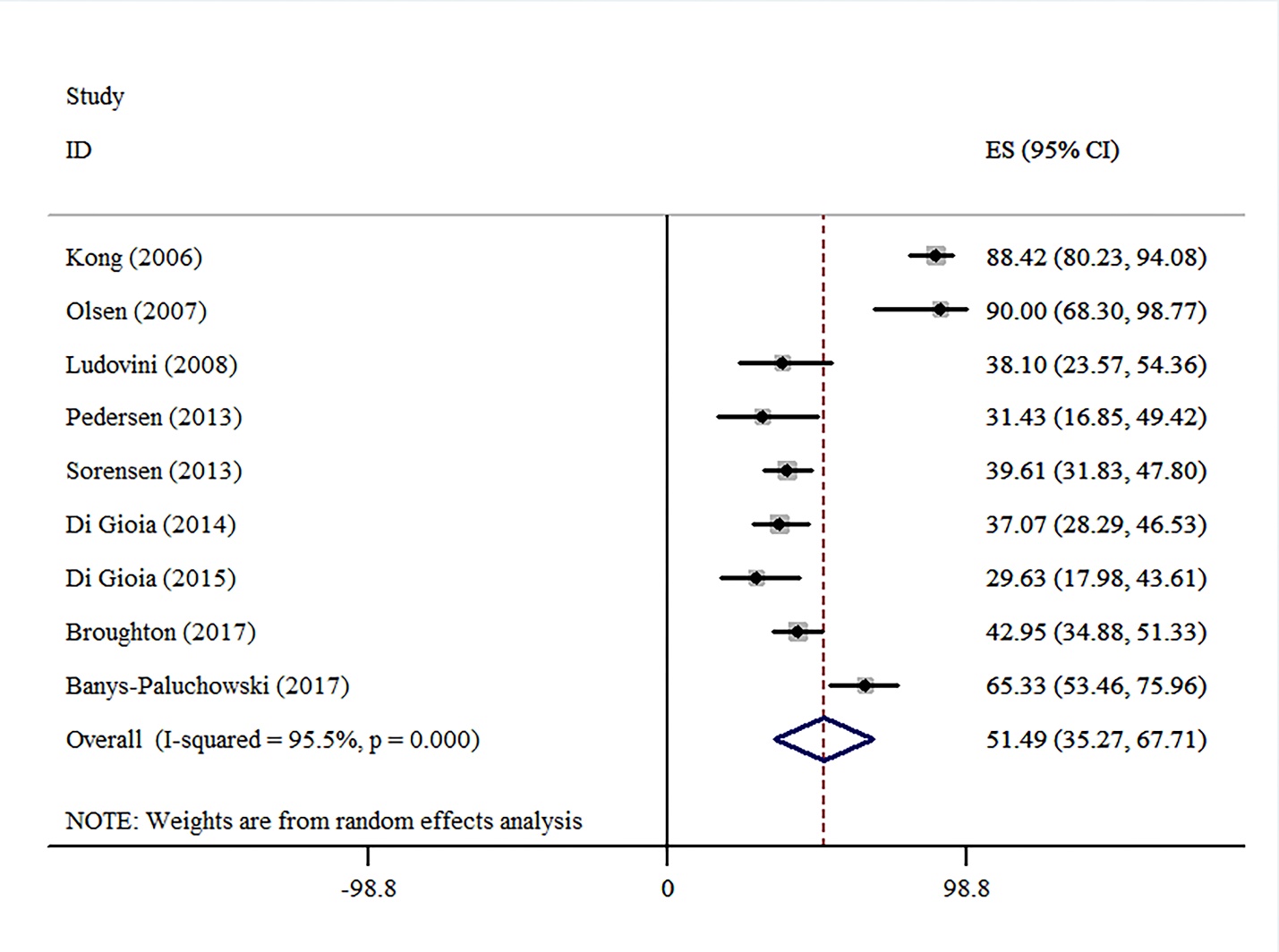


Supplementary Figure 4. Forest plot for sensitivity analysis of serum-HER2 sensitivity


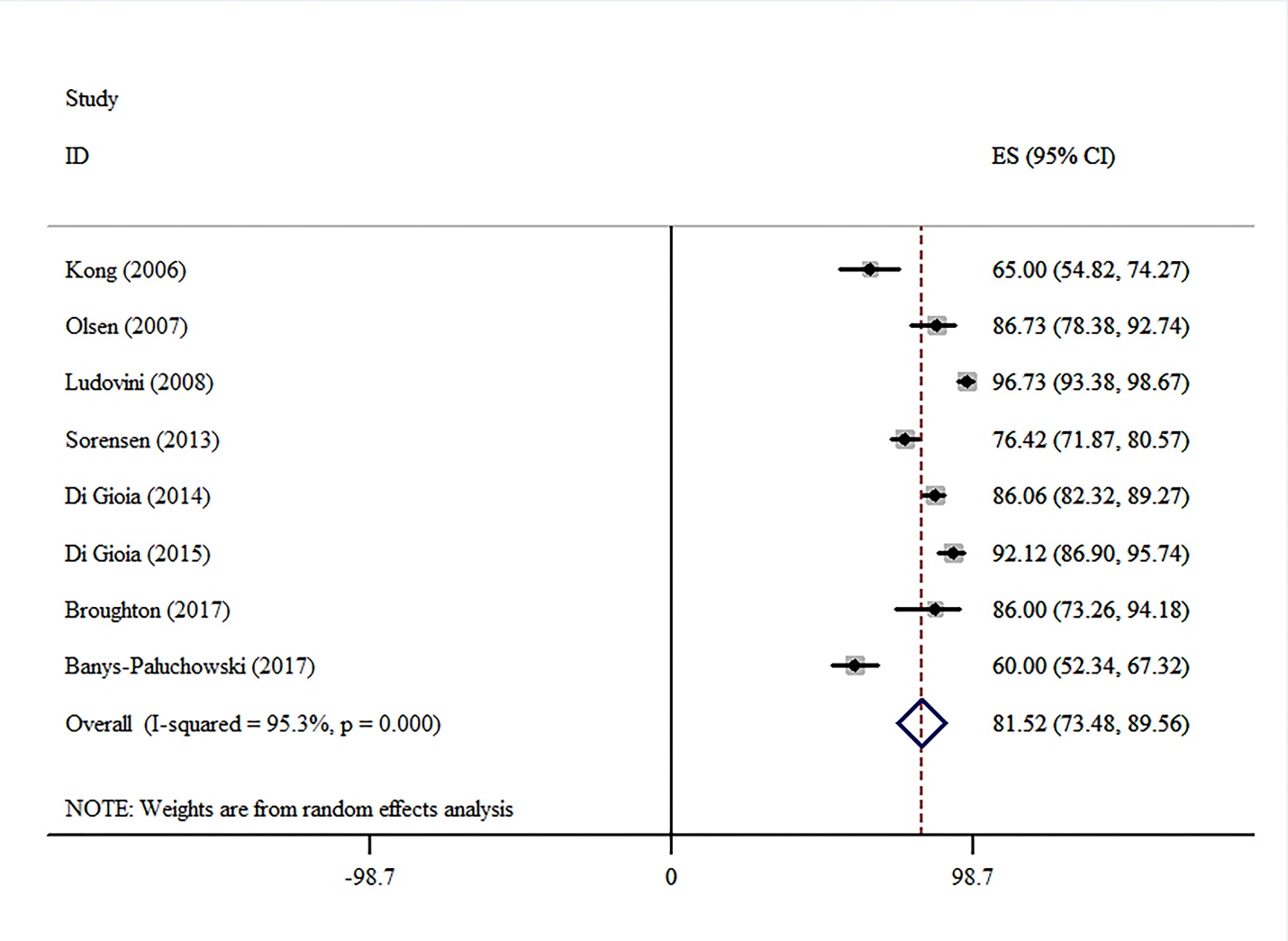


Supplementary Figure 5. Forest plot for sensitivity analysis of serum-HER2 specificity


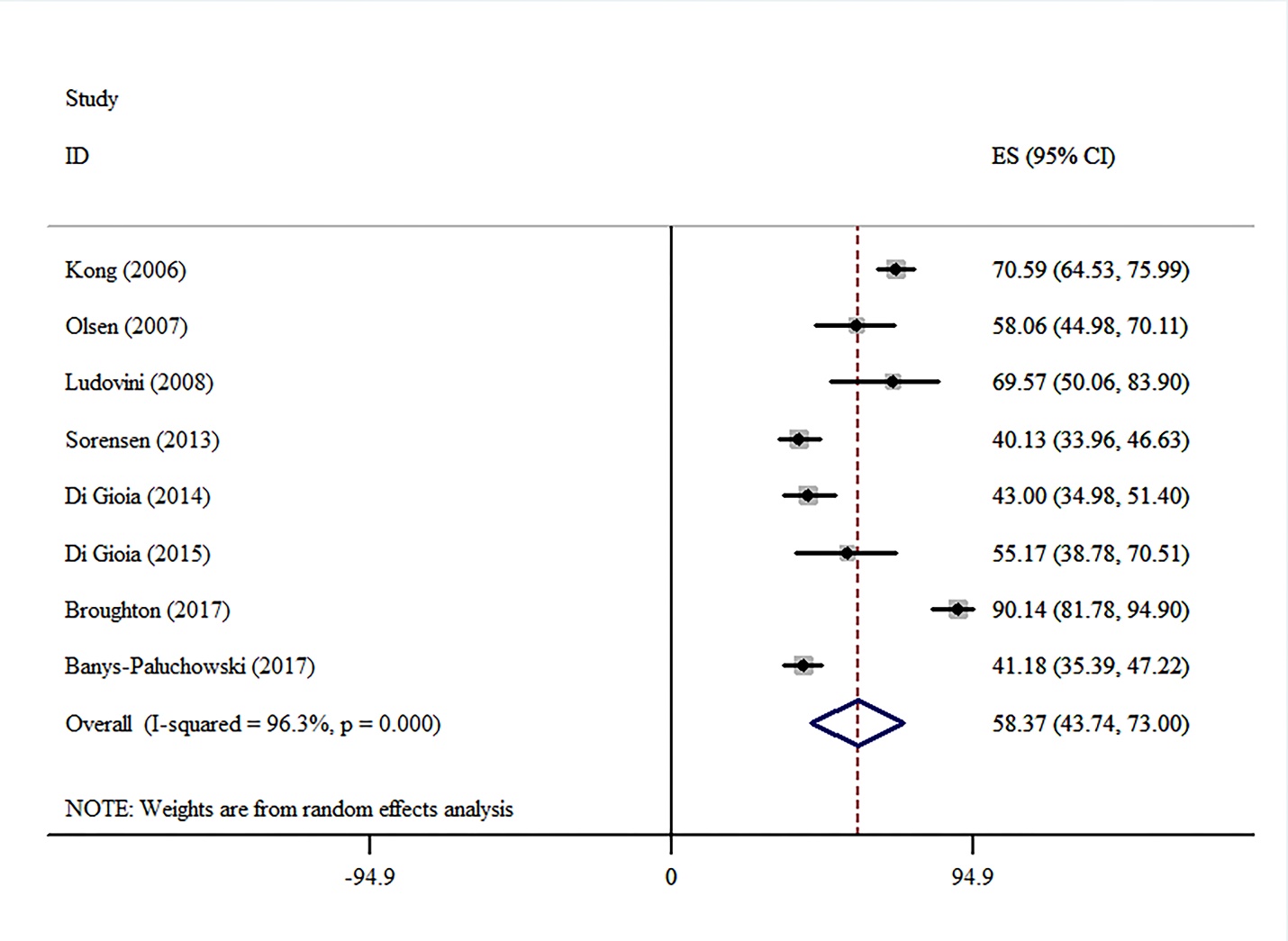


Supplementary Figure 6. Forest plot for sensitivity analysis of serum-HER2 Positive Predictive Value


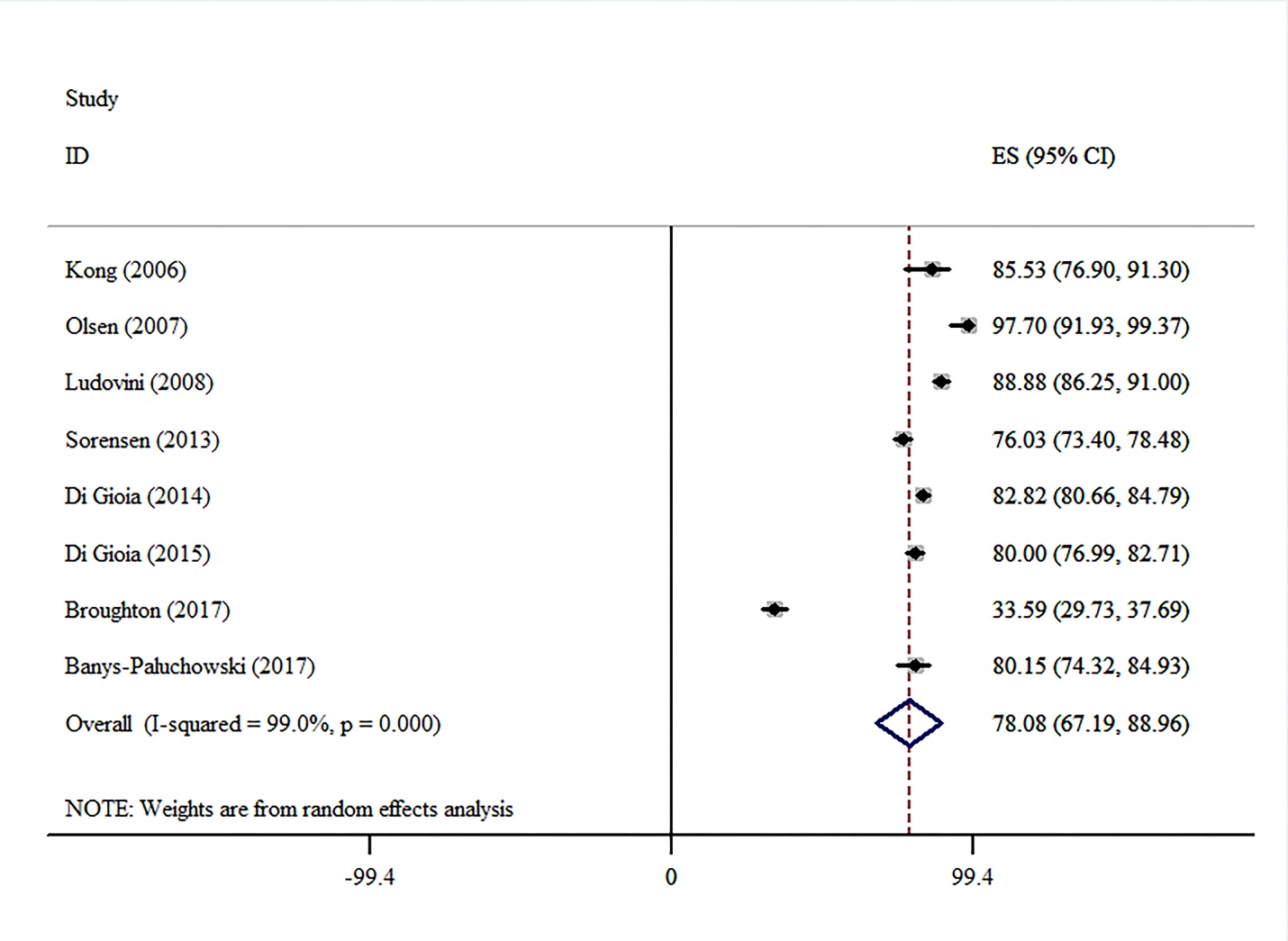


Supplementary Figure 7. Forest plot for sensitivity analysis of serum-HER2 Negative Predictive Value


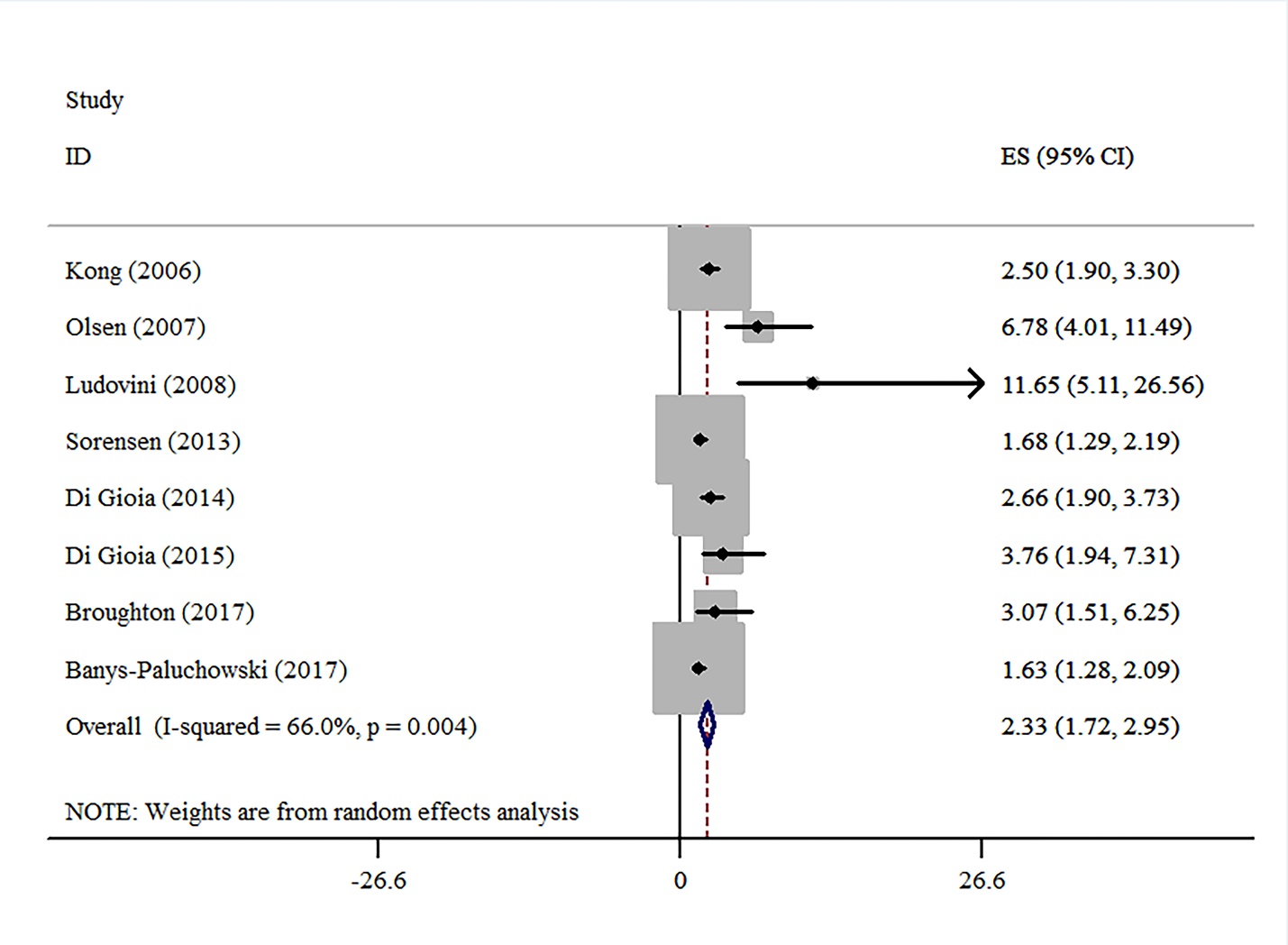


Supplementary Figure 8. Forest plot for sensitivity analysis of serum-HER2 Positive Likelihood Ratio


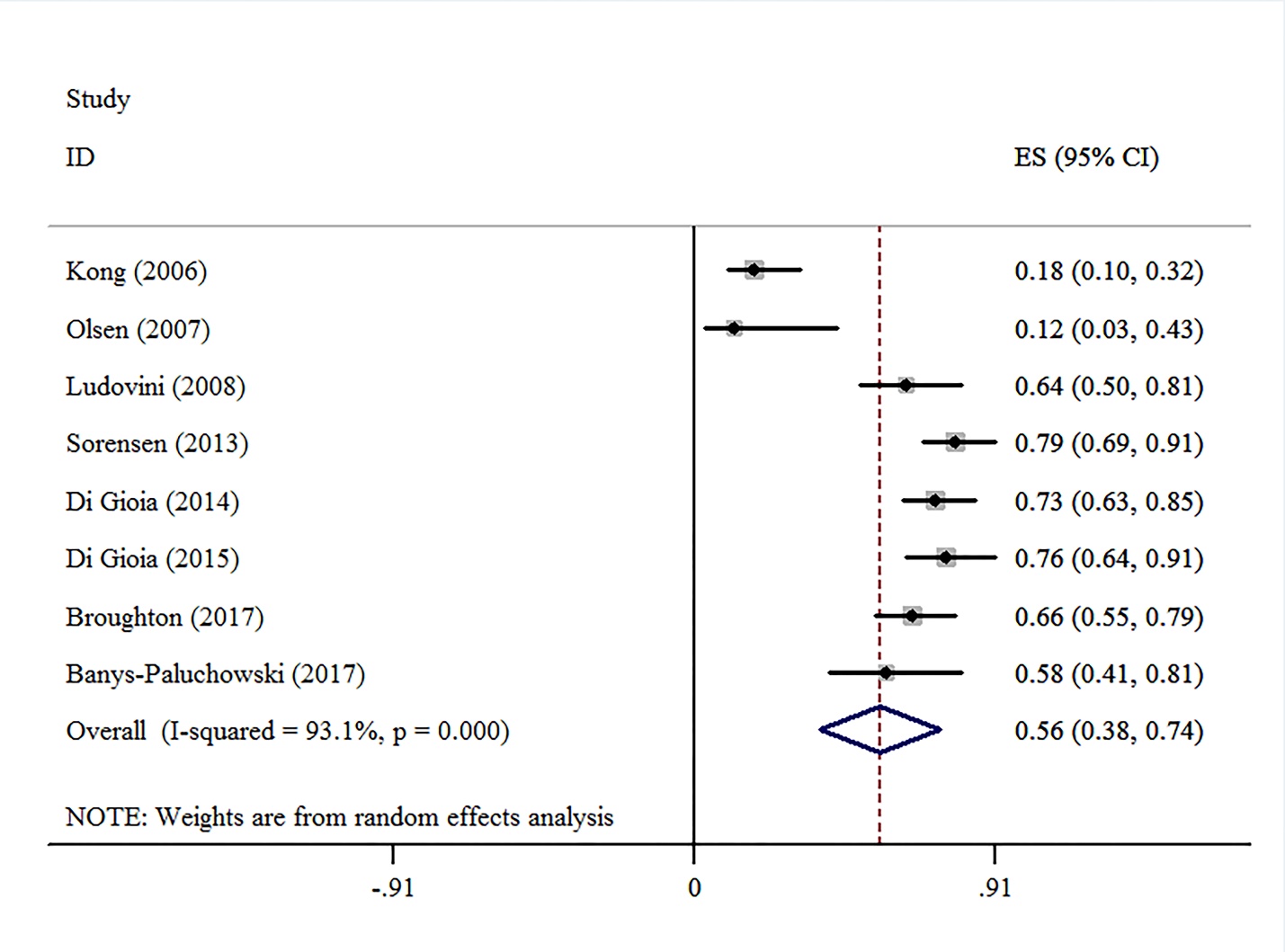


Supplementary Figure 9. Forest plot for sensitivity analysis of serum-HER2 Negative Likelihood Ratio


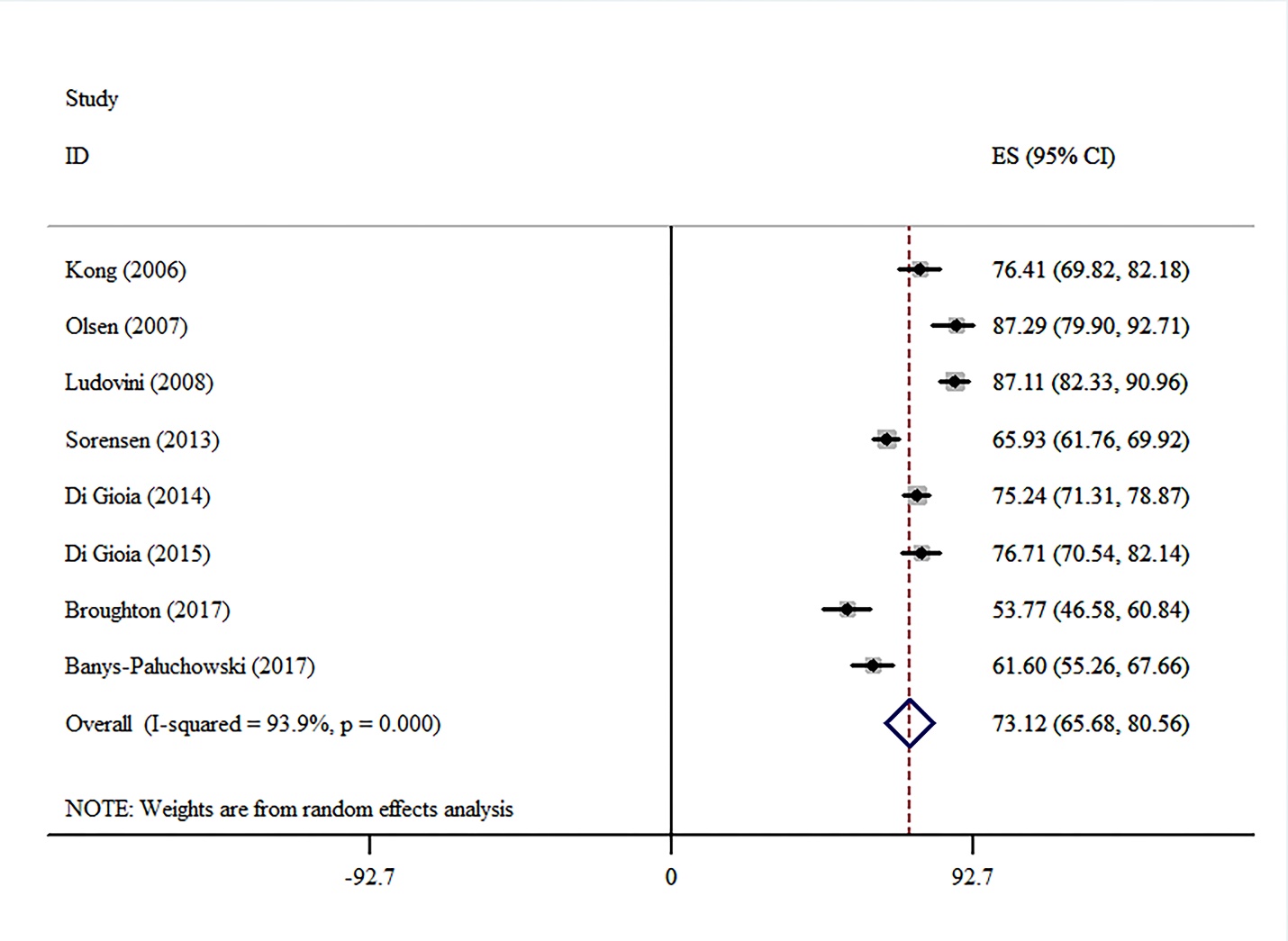


Supplementary Figure 10. Forest plot for sensitivity analysis of serum-HER2 accuracy
